# The QseC Adrenergic Signaling Cascade in Enterohemorrhagic *E. coli* (EHEC)

**DOI:** 10.1371/journal.ppat.1000553

**Published:** 2009-08-21

**Authors:** David T. Hughes, Marcie B. Clarke, Kaneyoshi Yamamoto, David A. Rasko, Vanessa Sperandio

**Affiliations:** 1 Department of Microbiology, University of Texas Southwestern Medical Center, Dallas, Texas, United States of America; 2 Department of Biochemistry, University of Texas Southwestern Medical Center, Dallas, Texas, United States of America; 3 Department of Agricultural Chemistry, Kinki University, Nakamachi, Nara, Japan; 4 Institute for Genome Sciences & Department of Microbiology and Immunology, University of Maryland School of Medicine, Baltimore, Maryland, United States of America; The Rockefeller University, United States of America

## Abstract

The ability to respond to stress is at the core of an organism's survival. The hormones epinephrine and norepinephrine play a central role in stress responses in mammals, which require the synchronized interaction of the whole neuroendocrine system. Mammalian adrenergic receptors are G-coupled protein receptors (GPCRs); bacteria, however, sense these hormones through histidine sensor kinases (HKs). HKs autophosphorylate in response to signals and transfer this phosphate to response regulators (RRs). Two bacterial adrenergic receptors have been identified in EHEC, QseC and QseE, with QseE being downstream of QseC in this signaling cascade. Here we mapped the QseC signaling cascade in the deadly pathogen enterohemorrhagic *E. coli* (EHEC), which exploits this signaling system to promote disease. Through QseC, EHEC activates expression of metabolic, virulence and stress response genes, synchronizing the cell response to these stress hormones. Coordination of these responses is achieved by QseC phosphorylating three of the thirty-two EHEC RRs. The QseB RR, which is QseC's cognate RR, activates the flagella regulon which controls bacteria motility and chemotaxis. The QseF RR, which is also phosphorylated by the QseE adrenergic sensor, coordinates expression of virulence genes involved in formation of lesions in the intestinal epithelia by EHEC, and the bacterial SOS stress response. The third RR, KdpE, controls potassium uptake, osmolarity, and also the formation of lesions in the intestine. Adrenergic regulation of bacterial gene expression shares several parallels with mammalian adrenergic signaling having profound effects in the whole organism. Understanding adrenergic regulation of a bacterial cell is a powerful approach for studying the underlying mechanisms of stress and cellular survival.

## Author Summary

Bacterial cells respond to the human stress hormones epinephrine (adrenaline) and norepinephrine (noradrenaline). These hormones are sensed by a bacterial receptor named QseC, which is a sensor kinase in the membrane that increases its autophosphorylation upon binding to these host signals. In addition to recognizing these signals, QseC also responds to a bacterial hormone-like molecule named autoinducer-3 (AI-3) that is produced by the human intestinal microbial flora. In this manuscript we have mapped genetically and biochemically the QseC signaling cascade in the deadly pathogen enterohemorrhagic *E. coli* (EHEC) O157:H7. EHEC uses this signaling system to activate expression of virulence genes. We show that the QseC signaling cascade is very complex so it can precisely modulate when different virulence traits are expressed. Because these sensor kinases are being evaluated as drug targets, a profound understanding of this signaling pathway is important for the development of novel therapeutic strategies to combat bacterial infections.

## Introduction

The survival of an organism lies within its intrinsic ability to detect and efficiently respond to stress cues. Stress responses play a key role in adaptation to environmental, psychosocial, and physical insults. Hence it comes as no surprise that stress responses require synchronization and coordination of an organism's resources to ensure that metabolic substrates are available to meet the increasing energy demands of an effective stress response. Stress responses are generally termed “fight or flight” responses in higher animals, because they rely in the ability of an organism's to assess whether its better chance of survival relies on facing or avoiding an environmental insult. The hormones epinephrine and norepinephrine are at the core of stress responses [1].

In mammalian cells epinephrine and norepinephrine are recognized by GPCRs, which are membrane receptors coupled to heterotrimeric guanine-binding proteins (G-proteins). These proteins consist of three subunits α, β and γ. The binding of these signals to GPCRs result in a conformational change that activates the G-protein through the exchange of GDP for GTP. The activated G-protein dissociates from the receptor, the α, β, and γ subunits then dissociate and activate their intracellular targets. The GPCR specificity is controlled by the type of G-protein associated with the receptor. G-proteins are divided in four families according to their association with effector proteins. Three of these signaling pathways, Gα_s_, Gα_i_ and Gα_q_, have been extensively studied, with Gα_s_ activating adenylate cyclase, Gα_i_ inhibiting adenylate cyclase, and Gα_q_ activating phospholipoase C [1].

Most of the knowledge of epinephrine/norepinephrine-mediated signaling has been derived from studies in mammalian systems. However, although bacterial cells sense and respond to epinephrine and norepinephrine, the signaling pathways regulated by these mammalian hormones in bacteria have not been mapped [2],[3]. Bacteria do not express homologues of mammalian adrenergic receptors. These signals are sensed through histidine sensor kinases (HKs) [4],[5]. HKs constitute the predominant family of signaling proteins in bacteria. HKs usually act in concert with a response regulator (RR) protein constituting a two-component system. Upon sensing a defined environmental cue the HK autophosphorylates a conserved histidine residue, and then transfers this phosphate to an aspartate residue in the receiver domain of a cognate RR. The majority of the RRs are transcription factors, which are activated upon phosphorylation [6].

Two HKs, QseC and QseE, characterized in *E. coli* have been reported to sense epinephrine and norepinephrine [4],[5]. QseC binds to and increases its autophosphorylation in response to epinephrine, norepinephrine, and a bacterial signaling molecule termed autoinducer-3 (AI-3) [4]. QseE increases its autophosphorylation in response to epinephrine, phosphate and sulfate [5]. QseC acts upstream of QseE, given that transcription of *qseE* is activated by QseC [7]. The cognate RR for QseC is QseB [4], and the genes encoding this two-component system are co-transcribed constituting an operon [8]. The cognate RR for QseE is QseF, with the *qseF* gene also being co-transcribed with *qseE* within the same operon [8]. QseF, however, is also phosphorylated by four other non-cognate HKs: UhpB, BaeS, EnvZ and RstB [9]. QseC homologues exist in at least 25 bacterial species [10], while QseE homologues can only be found in enterics. This distribution of receptors may play a role in colonization or virulence with increased levels of epinephrine/norepinephrine.

The majority of the studies assessing adrenergic regulation of bacterial gene expression, have been conducted in bacteria that inhabit the human gastrointestinal (GI) tract [2],[4],[11],[12],[13],[14],[15]. Norepinephrine is present in the GI tract, being synthesized by adrenergic neurons of the enteric nervous system (ENS) [16]. Epinephrine is synthesized in the central nervous system and the adrenal medulla, and reaches the intestine in a systemic manner after being released into the bloodstream [17]. Norepinephrine is found at a nanomolar range in sera, while it is at a micromolar range in the intestine [18]. Both hormones have important roles in intestinal homeostasis regulating peristalsis, blood flow, chloride and potassium secretion [17],[19]. Both epinephrine and norepinephrine are recognized by the same adrenergic GPCRs in mammalian cells, and the ligand-binding site for these hormones is largely similar [20].

Enterohemorrhagic *Escherichia coli* (EHEC) O157:H7 is a GI pathogen that exploits adrenergic signaling to regulate virulence gene expression [2]. EHEC colonizes the human intestine and leads to the development of hemorrhagic colitis and hemolytic uremic syndrome (HUS). In the colon, EHEC forms attachment and effacement (AE) lesions on the intestinal epithelial cells, which cause extensive rearrangement of the host cell cytoskeleton resulting in the formation of a pedestal-like structure underneath the bacterial cell [21]. The genes required for AE lesion formation are located in the chromosomal pathogenicity island termed the locus of enterocyte effacement (LEE) [22]. The first operon in the island (named *LEE1*), encodes Ler, the master regulator of the LEE genes [23]. The remaining genes encode the type-three secretion system (TTSS) [24], which forms a syringe-like apparatus that the bacteria use to translocate effector molecules to the host cells. Many of these effectors mimic mammalian signaling proteins having profound effects in the host cell signal transduction culminating in diarrheal disease [25]. Seven of these effectors are encoded within the LEE region [25], while many others are scattered throughout the genome [26],[27]. The first secreted effector discovered outside of the LEE was NleA [28]. NleA is known to inhibit cellular protein secretion by disrupting mammalian COPII function and mutation of the *nleA* gene resulted in attenuation in mouse model of infection [28],[29]. EHEC also produces a potent Shiga toxin (Stx) that is responsible for the major symptoms of hemorrhagic colitis and HUS [30].

Expression of LEE, Shiga toxin and the flagella and motility genes in EHEC are regulated by the signals AI-3, epinephrine and norepinephrine through QseC [4],[10]. This regulation is important for EHEC virulence, given that *qseC* mutants are attenuated for infection in animal models of disease [4],[10]. QseC activates transcription of the *flhDC* genes, which encode the master regulators of the flagellar regulon, directly through QseB binding to the *flhDC* promoter. Importantly, this interaction is dependent on QseB's phosphorylation state [31], whereas, expression of the LEE and Shiga toxin genes are not regulated by QseB. Here we report a global analysis of EHEC gene expression in response to adrenergic signals, and map the QseC signaling cascade. In this study we unravel the adrenergic response of a bacterial cell at the genetic and biochemical levels, and demonstrate that adrenergic signaling has a profound effect on cell homeostasis, cell-to-cell signaling, and bacterial pathogenesis.

## Results

### Global assessment of QseC gene regulation in EHEC

**Figure 1:**
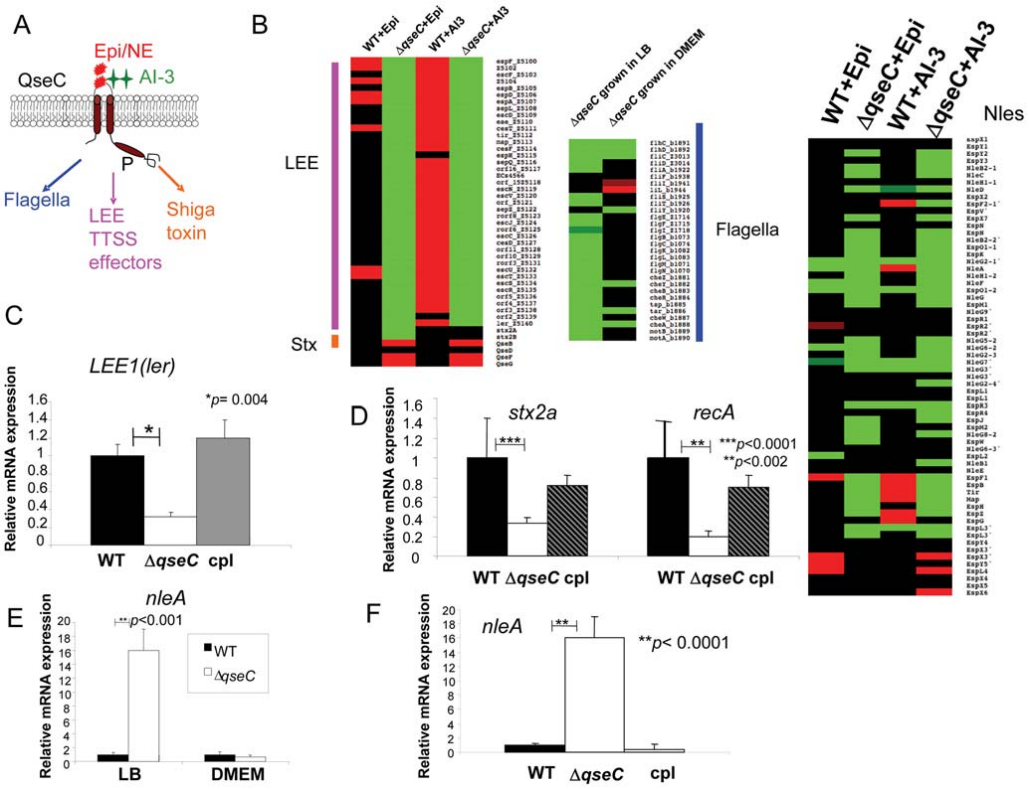
QseC regulates multiple virulence factors. (A) Schematic representation of QseC responding to epinephrine/norepinephrine and AI-3 and regulating multiple virulence factors (B) Heat maps from microarray analysis representing the effects of epinephrine and AI-3 on WT EHEC and Δ*qseC*, differential regulation of the LEE genes, the flagellar genes and the non-LEE encoded secreted effectors are shown. Both WT and Δ*qseC* produce AI-3 in DMEM (OD_600_ 1.0), hence these data reflect the transcriptome in the presence of AI-3 alone (WT+AI-3 and Δ*qseC*+AI-3) or AI-3 plus epinephrine (WT+Epi, Δ*qseC*+Epi) (C) QPCR of *ler* in wt EHEC, Δ*qseC*, and Δ*qseC* complement strain grown in DMEM (OD_600_ 1.0) (in the presence of self produced AI-3) (D) QPCR of *stx2a* and *recA* in wt EHEC, Δ*qseC*, and Δ*qseC* complement strain grown in DMEM (OD_600_ 1.0) (in the presence of self produced AI-3) (E) QPCR of *nleA* in WT EHEC and Δ*qseC* in LB and DMEM (OD_600_ 1.0) (in the presence of self produced AI-3) (F) QPCR of *nleA* in wt EHEC, Δ*qseC*, and Δ*qseC* complement strain grown in DMEM (OD_600_ 1.0) (in the presence of self produced AI-3).

**Figure 2:**
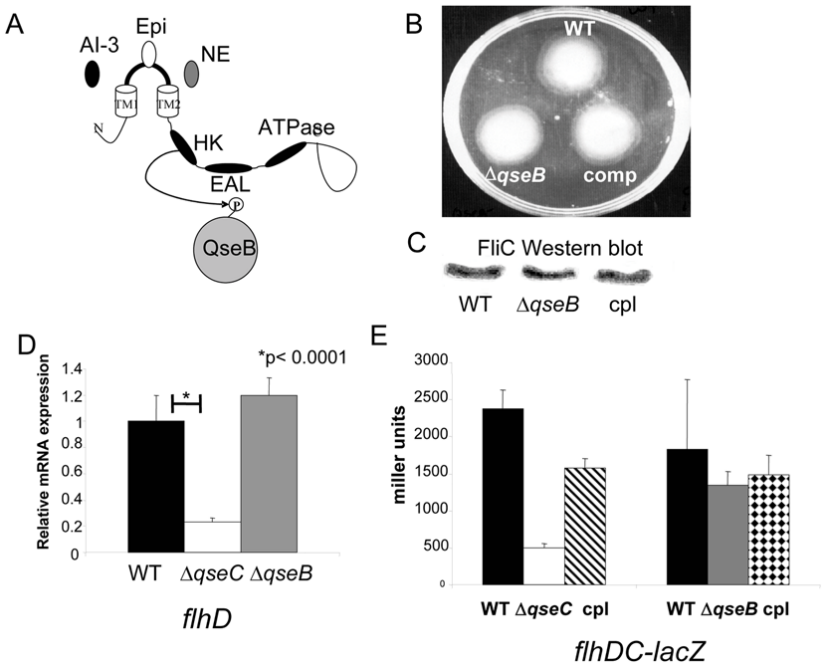
Δ*qseC* and Δ*qseB* do not have the same phenotype. (A) Schematic representation of QseC responding to the signals epinephrine/norepinephrine and AI-3 and transferring its phosphate onto its cognate response regulator QseB. (B) Motility plate of wt EHEC, Δ*qseB*, and the Δ*qseB* complement strain (complemented with plasmid pVS178, *qseBC* in pBAD33 [35]) (in the presence of self produced AI-3) (C) Western blot of FliC in wt EHEC, Δ*qseB*, and the Δ*qseB* complement strain (complemented with plasmid pVS178, *qseBC* in pBAD33) (in the presence of self produced AI-3) (D) QPCR of *flhD* in wt EHEC, Δ*qseC*, and Δ*qseB* in LB (OD_600_ 1.0) (in the presence of self produced AI-3) (E) β-galactosidase assay of the *flhDC* promoter controlling *lacZ* expression in wt EHEC, Δ*qseC*, the Δ*qseC* complement strain, Δ*qseB*, and the Δ*qseB* complement strain (complemented with plasmid pVS178, *qseBC* in pBAD33) in LB (OD_600_ 1.0) (in the presence of self produced AI-3).

We had previously reported that inactivation of the *qseC* gene results in reduced flagella expression and motility, and reduced auto-activation [8],[31]. To further characterize the role of QseC in EHEC, Affymetrix *E. coli* 2.0 microarrays were used to compare expression profiles of the WT and Δ*qseC* strains in the presence and absence of the signals AI-3 and epinephrine in Dulbecco's modified eagle media (DMEM), which is optimal for expression of the LEE genes, and LB, which is optimal for expression of the flagella regulon. These arrays contain ∼10,000 probe sets (array genes), covering all genes in the genomes of the two sequenced EHEC strains (EDL933 and Sakai), K-12 strain MG1655, uropathogenic *E. coli* (UPEC) strain CFT073, and 700 probes to intergenic regions (which can encode non-annotated small ORFs, or small regulatory RNAs). Expression data can be accessed using accession number (GSE15050) at the NCBI GEO database. During growth in LB, 126 probe sets were down-regulated (28 specific to EHEC), and 708 were up-regulated (232 EHEC specific) in the *qseC* mutant (Table 1). The majority of the genes with an altered profile were derived from the *E. coli* K-12 strain MG1655 (68%), which represent a common *E. coli* backbone conserved among all *E. coli* pathovars [32]. Many of these genes are associated with metabolism, and they also include the flagella regulon (Figure 1B and Figure 2D and 2E). The EHEC specific genes (32%) include several prophage-encoded genes and *stxAB* encoding Shiga toxin. These studies revealed that QseC not only activates transcription of the flagella regulon, but also of the genes encoding Shiga toxin.

**Table 1:** Comparison of 86-24 (WT) and the qseC mutant under different growth conditions.

	Increased	Marginal Increased	Decreased	Marginal Decreased	No Change
*qseC*-LB	708	130	126	112	9132
*qseC*-AI-3	106	562	273	206	9061
*qseC*-Epi	70	432	311	224	9171

Increased and decreased are at least two fold changes in the expression levels.

Marginally Increased or decreased are changes that are either less than two fold or designated as “marginally increased or decreased” by the Affymetrix analysis software GCOSv1.4.

Comparisons of *qseC*-AI3 to WT-AI3 were performed from RNA harvested from strains grown in DMEM (OD_600_ 1.0) (in the presence of self produced AI-3). Comparisons of *qseC*-Epi to WT-Epi were performed from RNA harvested from strains grown in DMEM (OD_600_ 1.0) (in the presence of self produced AI-3) with 10 µM epinephrine.

Transcriptome comparisons between WT and the *qseC* mutant grown in DMEM, a condition conducive to LEE and virulence gene expression, in the presence of AI-3 alone (both WT and the *qseC* mutant produce AI-3 when grown to late exponential phase in DMEM) or AI-3 plus epinephrine also revealed a global role for QseC regulation of virulence genes (Table 2). In the presence of AI-3 alone, expression of 106 genes was increased and 273 decreased in the *qseC* mutant compared to WT. In the presence of AI-3 plus epinephrine expression of 70 genes was increased and 311 decreased in the *qseC* mutant compared to WT. AI-3 and epinephrine have been reported to act as agonistic signals [33]. This agonistic relationship in signaling can be further illustrated by the observation that while AI-3 is only sensed through QseC, epinephrine is sensed by both QseC and QseE [4],[5]. However, it is worth mentioning that QseC acts upstream of QseE, given that transcription of *qseE* is activated by QseC [7]. These data suggest that both signals tend to activate global gene expression in a *qseC*-dependent fashion more frequently than repress expression. Among the genes activated in a *qseC*-dependent manner are the LEE (through activation of *ler* transcription, within the *LEE1* operon, encoding the Ler activator of all other LEE genes) and *stxAB* (Shiga toxin) genes (Figure 1B, 1C and 1D). The genes encoding Stx are located within the late genes of a λ- bacteriophage and are transcribed when the phage enters its lytic cycle upon induction of an SOS response in the bacterial cell [34]. Upon the induction of an SOS response, *recA* is upregulated and cleaves the λ cI repressor allowing transcription of the middle and late genes to proceed, and together with them the *stxAB* genes. QseC-induction of *stxAB* transcription occurs through induction of *recA* expression (Figure 1D), suggesting that QseC mediates SOS induction in bacterial cells. In addition to activating expression of the LEE-encoded TTSS, the majority of the genes encoding effectors translocated through this TTSS are also regulated by QseC (Figure 1B). Of note, transcription of the gene encoding the NleA effector is strongly repressed by QseC in LB, while its expression is slightly (non-statistically significant) decreased in the *qseC* mutant in DMEM (Figure 1E and 1F).These analyses confirmed QseC's activation of the flagellar genes and revealed several new regulatory targets, including: LEE (through *ler*), *nleA*, genes of the SOS response and Shiga toxin. Altogether, these data suggest that QseC is at the top of the signaling cascade activated by AI-3, epinephrine and norepinephrine, initiating regulation of all EHEC virulence genes.

**Table 2:** Pathovar distribution under different growth conditions.

	MG1655	EDL933	Sakai	CFT073	Intergenic
***qseC*** **-LB**					
Decreased	56	25	3	34	3
Marg_decreased	45	35	2	24	3
Increased	266	194	38	121	83
Marg_increased	56	37	7	14	16
No Change	3647	1496	323	2293	1192
***qseC*** **-DMEM-Epi**					
Decreased	75	144	23	44	25
Marg_decreased	115	62	10	12	5
Increased	36	7	5	14	2
Marg_increased	239	62	14	65	28
No Change	3605	1512	321	2351	1237
***qseC*** **-DMEM-AI3**					
Decreased	118	109	13	22	11
Marg_decreased	135	42	8	6	15
Increased	46	18	5	23	13
Marg_increased	245	156	30	59	49
No Change	3526	1462	317	2376	1209

Increased and decreased are at least two fold changes in the expression levels.

Marginally increased or decreased are changes that are either less than two fold or designated as “marginally increased or decreased” by the Affymetrix analysis software GCOSv1.4.

Comparisons of *qseC*-AI3 to WT-AI3 were performed from RNA harvested from strains grown in DMEM (OD_600_ 1.0) (in the presence of self produced AI-3). Comparisons of *qseC*-Epi to WT-Epi were performed from RNA harvested from strains grown in DMEM (OD_600_ 1.0) (in the presence of self produced AI-3) with 10 µM epinephrine.

### The QseC signaling transduction pathway

Through QseC, EHEC senses AI-3, epinephrine and norepinephrine to activate flagella and motility, AE lesion formation and Shiga toxin expression. Given that these are expensive biological processes that have to occur in concert, the kinetics of expression of these genes has to be exquisitely fine-tuned. We have previously reported that a Δ*qseC* EHEC had reduced motility, expressed less flagella, and presented reduced transcription of the flagella regulon [35]. The cognate RR of the QseC HK is QseB, which is phosphorylated at a conserved aspartate residue by QseC [4] (Figure 2A). In this study we deleted the cognate response regulator *qseB*. Since we had previously shown that QseC regulated the flagellar genes through a direct interaction of QseB and the *flhDC* promoter (FlhDC are the master activators of the flagella regulon) [31], we hypothesized that mutation of *qseB* would result in decreased motility. However, a Δ*qseB* mutant has no motility defect (Figure 2B), and expresses flagella at the same levels as the WT strain (Figure 2C). To confirm these results, we assessed transcription of *flhD* by real-time RT-PCR in WT, Δ*qseC*, and Δ*qseB* mutants. Relative expression levels of *flhD* in these three strains indicated that transcription of *flhD* is decreased in Δ*qseC* but is unaltered in Δ*qseB* (Figure 2D). We then performed β-galactosidase assays with the −900 to +50 bp region of the *flhDC* promoter fused to a promoterless *lacZ* gene as a reporter. We found that in Δ*qseC* there was five-fold less β-galactosidase activity as compared to WT (Figure 2E), but there was no difference in β-galactosidase activity between the WT and Δ*qseB*. Because QseB and QseC constitute a cognate two-component system, we expected that the *qseC* and *qseB* mutants would have similar phenotypes. However, while the *qseC* mutant has decreased motility and expression of the flagellar regulon, the *qseB* mutant shows similar levels of *flhDC* expression and motility as the WT strain. These results led us to develop two potential hypotheses for the differential effects of knocking an HK (QseC) and its cognate RR (QseB) on *flhDC* transcription. First, QseB can bind to different DNA sequences according to its phosphorylation state, acting as a repressor or activator depending on which site it is bound to. Second, QseC could be a promiscuous HK and can phosphorylate non-cognate RRs that acts on the *flhDC* promoter.

**Figure 3:**
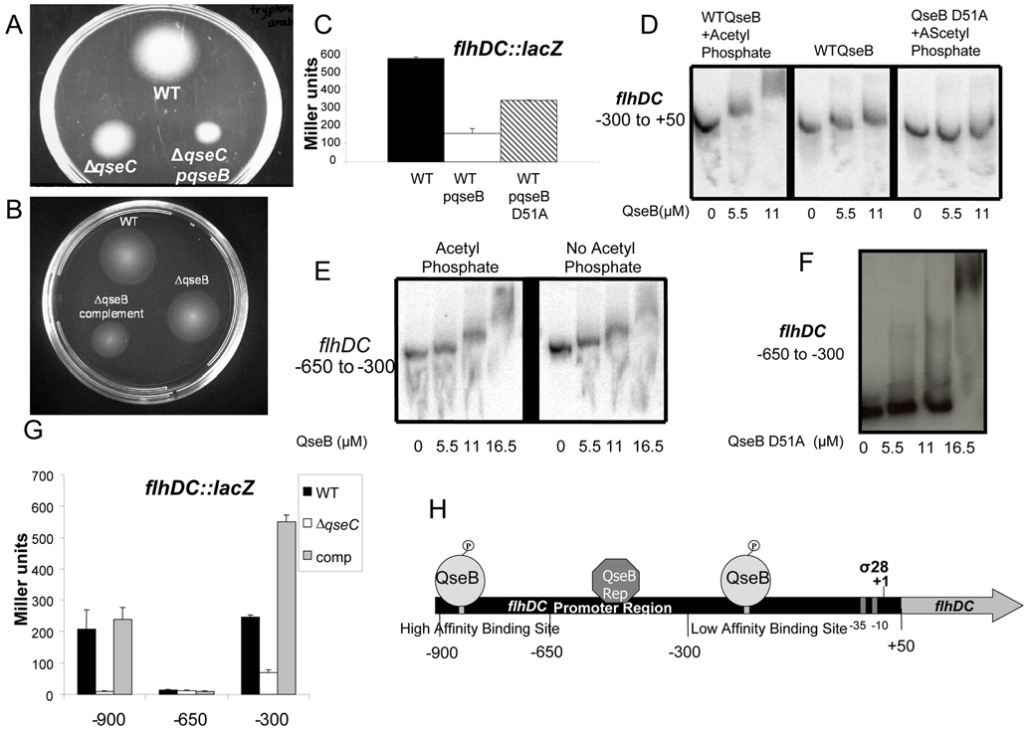
QseB differentially regulates *flhDC* based on its phosphorylation state. (A) Motility plate of wt EHEC, Δ*qseC*, and Δ*qseC* overexpressing *qseB* (in the presence of self produced AI-3) (B) Motility plate of wt EHEC, Δ*qseB*, and Δ*qseB* overexpressing *qseB* (in the presence of self produced AI-3) (C) β-galactosidase assay of the *flhDC* promoter controlling *lacZ* expression in wt EHEC and in wt EHEC overexpressing *qseB* and the QseB D51A mutant in LB (OD_600_ 1.0) (in the presence of self produced AI-3) (D) EMSA of the −300 bp to +50 bp region of the *flhDC* promoter with QseB and the phosphor-donor acetyl phosphate, QseB, and the nonphosphorylatable QseB D51A with the phosphor-donor acetyl phosphate (E) EMSA of the −50 bp to +50 bp region of the *flhDC* promoter with QseB in the presence and absence of the phosphor-donor acetyl phosphate (F) EMSA of the −650 bp to +50 bp region of the *flhDC* promoter with the nonphosphorylatable QseB D51A (G) Nested deletion β-galactosidase analysis of the *flhDC* promoter (−900 bp to +50 bp, −650 bp to +50 bp, and −300 bp to +50 bp) controlling *lacZ* expression in wt EHEC, Δ*qseC*, the Δ*qseC* complement strain in LB (OD_600_ 1.0) (in the presence of self produced AI-3) (H) QseB binding sites on the *flhDC* promoter.

To test the first hypothesis we overexpressed QseB in a Δ*qseC* background. We assumed that this strain would have an overabundance of unphosphorylated QseB. We found that this strain was less motile than Δ*qseC*, indicating that unphosphorylated QseB can act as a repressor of the flagellar gene expression (Figure 3A). We also complemented the Δ*qseB* strain with a plasmid expressing QseB, and observed that the complemented strain had decreased motility; again suggesting that overabundance of unphosphorylated QseB has a repressive role in motility (Figure 3B). However, when we complemented the Δ*qseB* strain with a plasmid expressing *qseBC* (Figure 2), we did not observe any differences in motility, probably because the levels of QseB and QseC were balanced in this strain. Next, we overexpressed *qseB*, in a strain containing the −900 to +50 bp region of the *flhDC* promoter upstream of a promoterless *lacZ*. We found that in the strain overexpressing *qseB* there was a five-fold decrease in β-galactosidase activity (Figure 3C). We also observed decreased *flhDC* transcription in a strain overexpressing a QseB site-directed mutant (QseB D51A) that cannot be phosphorylated (the conserved aspartate phosphorylated residue has been changed to an alanine) (Figure 3C), further indicating that an abundance of unphosphorylated QseB represses expression of *flhDC*.

We had previously shown that QseB can bind to two regions of the *flhDC* promoter, −300 to +50 bp and −900 to −650 bp [31]. We demonstrated that this binding required QseB to be phosphorylated [31] (Figure 3C), which can be achieved by providing a small phosphate donor, acetyl phosphate, to QseB *in vitro*. QseB will only bind to the −300 to +50 bp *flhDC* region in the presence of acetyl phosphate (Figure 3D), and the QseB D51A mutant is also unable to bind to this region of *flhDC* (Figure 3D). We have discovered a new QseB binding site in the *flhDC* promoter from −650 to −300 bp to which QseB can bind in the absence of phosphorylation. QseB binds to this −650 to −300 bp site in the absence of acetyl phosphate, and QseB D51A can also bind to this site (Figure 3E and 3F). The presence of this new binding site provides further evidence for a dual role of QseB in the regulation of the *flhDC* promoter. At low signal concentration there is low QseC activation and thus low QseB phosphorylation. In this case only the −650 to −300 bp site of the *flhDC* promoter will be occupied by non-phosphorylated-QseB and this binding may lead to repression. When the signal is high the opposite is true. The −300 to +50 bp and −900 to −650 bp sites will be occupied by phosphorylated QseB and *flhDC* will be activated (Figure 3H). In further support of this model, a nested deletion analyses of the *flhDC* promoter fused to *lacZ* shows that the full length fusion (−900 to +50 bp) is activated by QseC (Figure 3G). This fusion contains all three QseB binding sites, and in the presence of QseC, phosphorylated QseB will occupy the activating sites from −950 to −650 bp and −300 to +50 bp, increasing transcription. In the −650 to +50 bp fusion, transcription of *flhDC* is repressed in the absence or presence of QseC, probably because of non-phosphorylated QseB binding to the −650 to −300 bp site, which represses *flhDC* transcription. Non-phospho-QseB binding to the −650 to −300 bp region is probably “locked” in the absence of the upstream (−900 to −650) site. When both upstream sites are removed (−300 to +50 bp fusion), phospho-QseB bound to this proximal site will activate *flhDC* transcription (Figure 3F). In the complete absence of QseB, as in a *qseB* null strain, there will be QseC-independent expression of *flhDC* transcription, without any repression or activation (de-repression) by QseB (Figure 2). These data indicate that regulation of *flhDC* transcription by QseC occurs through its cognate RR QseB, and that QseB plays a dual role in this regulation according to its phosphorylated state.

**Figure 4:**
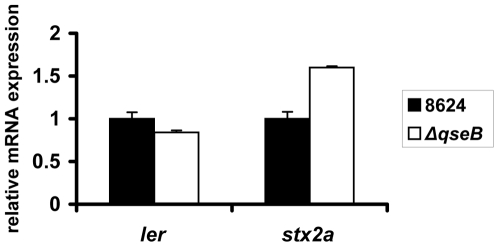
qPCR of *ler* and *stx2a* in wt EHEC and Δ*qseB* in DMEM (OD_600_ 1.0) (in the presence of self produced AI-3).

QseB, however, does not seem to play a role in QseC-dependent activation of LEE and *stxAB* transcription (Figure 4), suggesting that this regulation may occur through phosphorylation of other RRs. In addition to QseB there are at least 31 other RR in *E. coli* that could be activated via QseC [9]. There is minimal cross-talk (cross-phosphorylation) between different two-component systems ensuring faithful transmission of information through distinct signaling pathways [36],[37]. Indeed, the incidence of cross-phosphorylation between non-cognate HKs and RRs is low in *E. coli*, Yamamoto *et al*. showed that phosphorylation of non-cognate response regulators by HKs is rare and occurs in only 22 of 692 possible combinations [9]. However, in this same study, Yamamoto noticed that a distinct few HKs are more prone to also signal through non-congate RRs.

**Figure 5:**
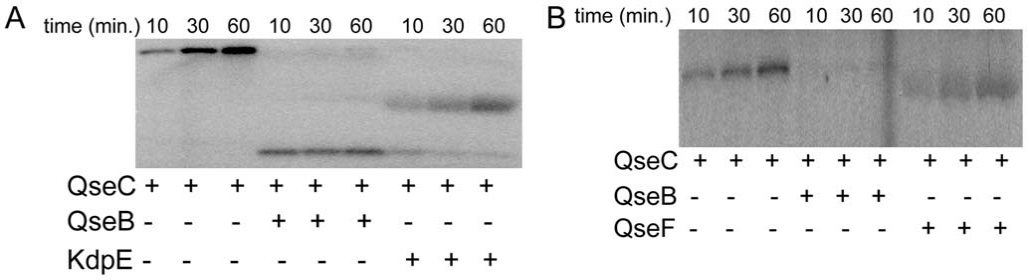
QseC phosphotransfers to the response regulators QseB, QseF and KdpE. (A) Autoradiograph of QseC autophosphorylation in lipid vesicles in the presence of 10 µM epinephrine and phosphotransfer onto QseB and KdpE (B) Autoradiograph of QseC autophosphorylation in lipid vesicles in the presence of 10 µM epinephrine and phosphotransfer onto QseB and QseF.

**Figure 6:**
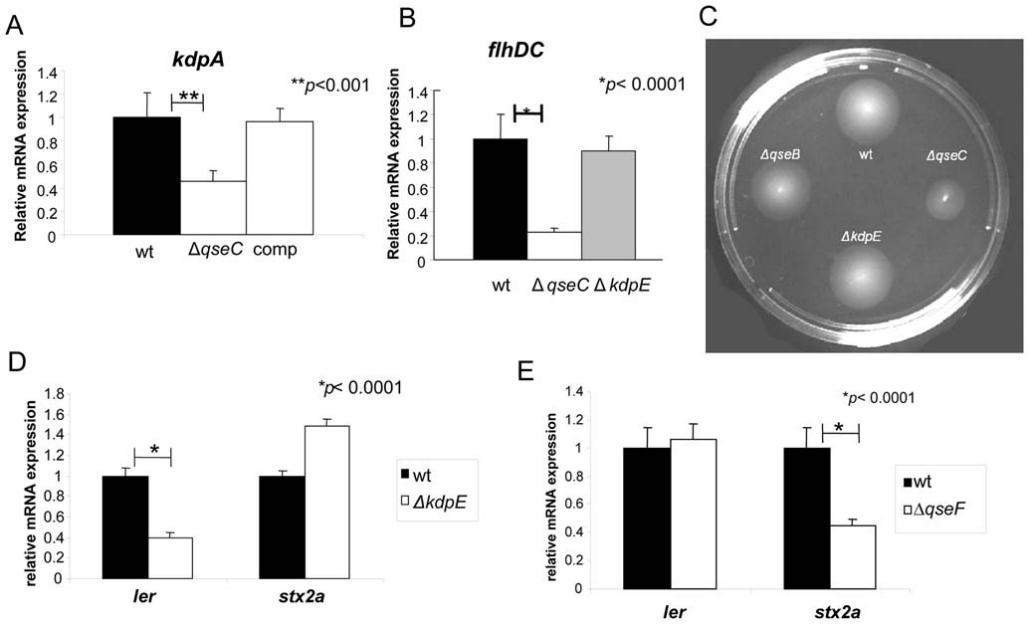
QseC, KdpE and QseF regulatory targets. (A) QPCR of *kdpA* in wt EHEC, Δ*qseC*, and Δ*qseC* complement strain in DMEM (OD_600_ 1.0) (in the presence of self produced AI-3) (B) QPCR of *flhD* in wt EHEC, Δ*qseC*, and Δ*kdpE* in DMEM (OD_600_ 1.0) (in the presence of self produced AI-3) (C) Motility plate of wt EHEC, Δ*qseB*, Δ*kdpE*, and Δ*qseC* (D) QPCR of *ler* and *stx2a* in wt EHEC and Δ*kdpE* in DMEM (OD_600_ 1.0) (in the presence of self produced AI-3) (E) QPCR of *ler* and *stx2a* in wt EHEC and Δ*qseF* in DMEM (OD_600_ 1.0) (in the presence of self produced AI-3).

We have previously reported that QseC autophosphorylates in response to AI-3, epinephrine and norepinephrine in an *in vitro* liposome assay and can phosphotransfer onto its cognate RR, QseB [4]. In order to test QseC's ability to phosphotransfer onto non-cognate RRs, we purified 31 *E. coli* RRs and performed phosphotransfer assays with QseC in liposomes. Of note all of these RRs were soluble and correctly folded upon purification, and have been previously shown by Yamamoto et al. to be active in phosphotransfer reactions with their cognate HKs [9]. Through this assay, we found only two additional QseC phosphorylation targets: KdpE and QseF (Table 3, Figure 5A and 5B). KdpE has been shown to regulate potassium uptake and medium osmolarity [38]. We found that *kdpA*, one of the genes regulated by KdpE, is also down-regulated in the Δ*qseC* (Figure 6A), indicating that cross-phosphorylation between QseC and KdpE results in QseC regulation of KdpE-dependent targets. To assess the contribution of KdpE to QseC's signaling transduction pathway, we deleted *kdpE* but found no motility defect (Figure 6C) or decreased *flhDC* expression (Figure 6B) in the *kdpE* mutant, indicating that KdpE is not regulating *flhDC*. When we assessed transcription of *ler* (LEE) and *stx*, we observed that KdpE activates transcription of the LEE genes, but not *stx*, suggesting that through the KdpE RR, QseC activates expression of the LEE genes (Figure 6D).

**Table 3:** QseC response regulator phosphotransfer table.

Response regulator	Phosphorylated by QseC
ArcA	No
AtoC	No
BaeR	No
BasR	No
CheB	No
CheY	No
CitB	No
CpxR	No
CreB	No
CusR	No
DcuR	No
EvgA	No
HydG	No
**KdpE**	**Yes**
NarL	No
NarP	No
NtrC	No
PhoB	No
PhoP	No
**QseB**	**Yes**
**QseF**	**Yes**
RcsB	No
RssB	No
RstA	No
TorR	No
UhpA	No
UvrY	No
YedW	No
YehT	No
YfhK	No
YhjB	No
YpdB	No
UvrY	No

Phosphotranfer studies were performed with purified QseC inserted in a liposome in the presence of 50 µM epinephrine and each purified response regulator. Yes indicates that QseC phosphotransfers to that response regulator (positive responses are bolded), No indicates the absence of phosphotransfer.

The second non-cognate RR phosphorylated by QseC, QseF, is responsible for aiding in AE lesion formation by activating expression of the phage-encoded gene *espFu* [7]. EspFu is a secreted effector, translocated to epithelial cells by the LEE-encoded TTSS, and it is involved in host actin nucleation and polymerization for AE lesion formation [39],[40]. QseF, however is not involved in regulation of LEE gene expression (Figure 6E) [7], nor in flagella and motility regulation [7]. However, a *qseF* knockout presented diminished expression of the *stx* gene (Figure 6E), suggesting that QseC activation of Shiga toxin expression occurs through the QseF RR. The QseF cognate HK is QseE [9], which is a second bacterial adrenergic receptor that senses epinephrine, phosphate and sulfate [11]. The addition of epinephrine to EHEC activates expression of *qseEF*, and this regulation is eliminated in the Δ*qseC* mutant, indicating that QseC activates transcription of *qseEF* [7]. Transcriptional regulation of *qseEF* by QseC, in addition to cross-phosphorylation of QseF by QseC and QseE may fine tune the timing for switching from motility, to AE lesion formation to Shiga toxin production during infection.

**Figure 7:**
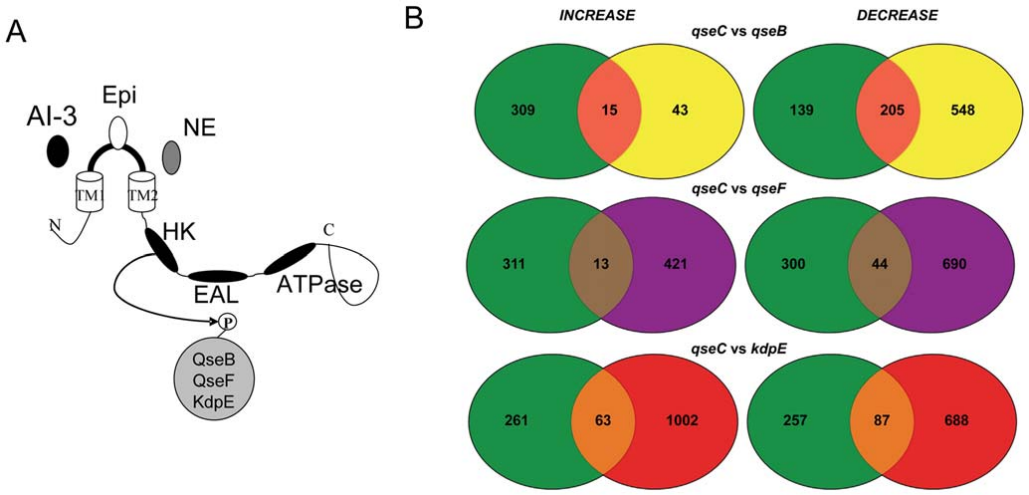
Regulatory overlap of QseC and its phosphorylation targets. (A) Schematic representation of QseC responding to the signals epinephrine/norepinephrine and AI-3 and transferring its phosphate onto QseB, KdpE, and QseF (B) Microarray analysis comparing Δ*qseC* to Δ*qseB*, Δ*kdpE*, and Δ*qseF* in DMEM (OD_600_ 1.0) (in the presence of self produced AI-3).

QseC phosphorylates three RRs: QseB, KdpE and QseF (Figure 7A). Through QseB the flagella regulon is regulated. KdpE activates expression of *ler*, and consequently of all LEE genes. QseF plays a role in inducing an SOS response and Shiga toxin production, as well as activating expression of *espFu* [7], which encodes an effector essential for AE lesion formation. To search globally which sets of QseC-dependent genes are regulated through each RR we performed transcriptome assays (GEO series GSE15050). These comparisons were performed with gene arrays hybridized with cDNA from RNA extracted from WT, Δ*qseC*, Δ*qseB*, Δ*kdpE* and Δ*qseF* strains grown in DMEM to an OD_600_ of 1.0, conditions known to yield maximal endogenous AI-3 production in these strains [41]. Given that AI-3 is only sensed through QseC, and QseC will phosphorylate in the presence of either AI-3 or epinephrine [4],[11], by working under these conditions we would detect only QseC-dependent genes. We avoided using epinephrine in these comparisons, because epinephrine is also sensed by the QseE HK [4],[11]. Transcription of 324 genes was increased, and 344 decreased in the Δ*qseC* mutant compared to WT (Figure 7B). Of the 324 genes increased in the Δ*qseC*, 15 were also increased in Δ*qseB*, 13 in Δ*qseF*, and 63 in Δ*kdpE* (Figure 7B). These data suggest that 91 of these 324 genes repressed by QseC are under the control of the QseB, KdpE and QseF RRs. These leaves 233 genes repressed through QseC unaccounted for. A possible explanation could be that these genes may be activated and repressed by QseB in a similar fashion to *flhDC* (Figure 3), and these genes would not appear as transcriptionally regulated through QseB using gene arrays. QseC activates transcription of 344 genes, with 205 being activated through QseB, 44 through QseF and 87 through KdpE (Figure 7B). These three RRs activate transcription of 336 of the 344 QseC-dependent genes, giving almost 100% coverage of QseC-activated genes.

## Discussion

Chemical signaling between cells underlies the basis of multi-cellularity. Although bacteria are unicellular, bacterial populations also utilize chemical signaling, through hormone-like compounds named autoinducers, to achieve cell-cell communication and coordination of behavior [42]. Chemical signaling is also essential for an organism to survive, successfully adapt to ever changing environments and protect themselves from insults, which can be collectively considered stress. Successful stress responses require energy input, and the coordination of many complex signaling pathways within the cell. Co-evolution of prokaryotic species and their respective eukaryotic host have exposed bacteria to hormones and eukaryotic cells to autoinducers. Therefore, it is not surprising that bacteria can respond to host hormones, and that some pathogenic species have high-jacked these signaling systems to promote disease states [43].

One example of a pathogen that senses host hormones to regulate virulence is EHEC [2]. Upon reaching the human colon, EHEC senses the autoinducer-3 (AI-3) produced by the microbial gastrointestinal flora, and epinephrine and norepinephrine produced by the host through the HK QseC [2],[4]. This signal transduction activates transcription of virulence genes in a coordinated fashion leading to the formation of AE lesions on intestinal cells by the locus of enterocyte effacement (LEE) genes, the flagella regulon for enhanced motility, and Shiga toxin production which is responsible for HUS. EHEC probably first encounters the AI-3 signal produced by the microbial flora that inhabits the intestinal lumen [2]. Because the infectious dose of EHEC is very low (estimated to be 50 CFUs) [21], it is unlikely that it responds to self-produced signal to initiate infection. Upon sensing AI-3, QseC initiates the signaling cascade that will activate the flagella regulon leading to swimming motility, which may aid EHEC to come closer to the intestinal epithelial layer. As EHEC approaches the epithelium and starts forming AE lesions it is probably then exposed to epinephrine and/or norepinephrine. Norepinephrine is synthesized within the adrenergic neurons of the enteric nervous system (ENS) that innervates the basolateral layer of the intestine [16]. Epinephrine is synthesized in the central nervous system (CNS) and in the adrenal medulla; it acts systemically after being released into the bloodstream, when it can reach the intestine [17]. AE lesion formation and the commencement of bloody diarrhea may increase EHEC exposure to epinephrine and norepinephrine, further upregulating expression of virulence genes in EHEC. This coordinated regulation involves a number of two-component regulatory systems composed of HKs and RRs that result in cascades of gene expression.

Recognition of AI-3/epinephrine/NE by QseC can be specifically blocked by the administration of the α-adrenergic antagonist phentolamine [4], and a synthetic compound called LED209 [10]. Using two different rabbit infection models it has been demonstrated that QseC plays an important role in pathogenesis *in vivo*, since *qse*C mutants were attenuated for virulence in these animals [4],[10]. Recently, a novel two-component system, the QseEF system [7], where QseE is the HK and QseF is the RR was shown to also regulate virulence in EHEC. QseE can also respond to the host hormone epinephrine like QseC, but in contrast, does not sense the bacterial signal AI-3. QseE is downstream from QseC in this signaling cascade, given that *qseEF* transcription is activated by epinephrine via QseC. The QseEF system is not involved in regulation of flagella and motility, but plays an important role in activating genes necessary for AE lesion formation [7] and also activates expression of Shiga toxin (Figure 6).

The AI-3/epinephrine/NE signaling system is not restricted to EHEC. *In silico* analysis showed homologues of QseC in other bacterial species such as *Salmonella* sp, *Shigella flexneri*, *Francisella tularensis*, *Haemophilus influenzae*, *Erwinia carotovora*, and many others [10]. *In vivo* studies provided evidence that the QseC HK is important in *Salmonella typhimurium* [10],[44] and *Francisella tularensis* [45] pathogenesis, since *qseC* mutants of these strains are attenuated in animal models of infection and *in vivo* inhibition of QseC by LED209 results in attenuation of infection by these organisms [10].

**Figure 8:**
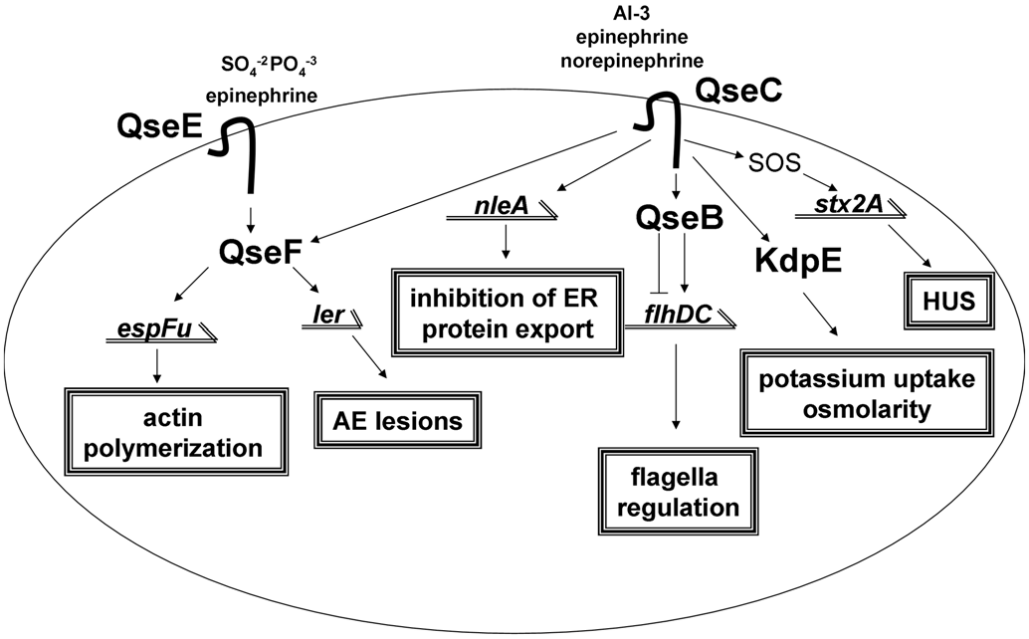
Model of the QseC and QseE signaling cascades in EHEC.

Because QseC is central for sensing adrenergic signals, and the effect these signals have in basic biological processes, a complete understanding of the QseC signaling transduction pathway in bacteria will offer clues on how eukaryotic stress responses affect a prokaryotic cell. We demonstrate that QseC acts promiscuously through three RRs (Figures 5 and 7) to initiate a complex signaling cascade that affects both metabolism and pathogenesis (Figure 8). QseC controls the expression of all of these features, either directly or indirectly and must be considered to be at or near the top of the signaling cascade. The fact that more that one kinase can activate multiple response regulators suggests that there is a hierarchy of signaling, beginning with QseC. It is currently unclear if the regulation by the associated HK and RR overrides the signal employed by a non-cognate HK or if they work in synergy to amplify the initial signal. This additional level of control may be the fine-tuning that is observed in EHEC where the motility, formation of lesions and secretion of toxin must be exquisitely choreographed to have an effective infection occur.

An additional level of complexity included in this signaling cascade is that QseB, binds to different sites in the target promoters according to its phosphorylation state (Figure 3). This allows further modulation of gene expression by the spatial arrangement of these sites in the regulatory region of genes, allowing the same RR to both repress and activate transcription of the same gene. In the non-activated form (non-phosphorylated) QseB forms an additional regulatory barrier to the expression of *flhDC*. Only under conditions where QseB is both phosphorylated and in sufficient concentration is there full activation of the flagella regulon. Thus this two-step process provides additional levels of control for this energetically expensive appendage. These types of mechanisms ensure that only under conditions which are favorable the resources are devoted to this response. The DNA binding domain of QseB shares similarities with the DNA binding domain of the OmpR RR, which also recognizes different sites on DNA according to its phosphorylation state [46],[47].

Because epinephrine and norepinephrine exert a profound effect in the host physiology and immune system, the ability to sense these hormones by bacteria may facilitate gauging the fitness of the host. Inter-kingdom chemical signaling plays an important role in the relationships forged between bacteria and animals. Chemical communication within kingdoms has been studied for many decades, however, the interception of these languages between different kingdoms has been appreciated only more recently. As this field expands, more and more examples will be described, and many questions answered.

## Materials and Methods

### Strains and plasmids

All bacterial strains and plasmids utilized in this study are listed in Table S1. *E. coli* strains were grown aerobically in LB or DMEM (Invitrogen) medium at 37°C unless otherwise stated. Antibiotics were added at the following concentrations: 100 µg ml^−1^ ampicillin and 30 µg ml^−1^ chloramphenicol.

### Recombinant DNA techniques

Standard methods were used to perform plasmid purification, PCR, ligation, restriction digests, transformation and gel electrophoresis [48].

### Isogenic mutant construction

Construction of isogenic *kdpE* (DH11) and *qseB* (MC474) mutants was carried out as previously described [49]. Briefly, 86-24 cells containing pKD46 were prepared for electroporation. A *kdpE* PCR product was generated using primers kdpEλRed-F and kdpEλRed-R (Table S2) and pKD3 as a template and PCR-purified (Qiagen). A *qseB* PCR product was generated using primers qseBλRed-F and qseBλRed-R (Table S2) and pKD3 as a template and PCR-purified (Qiagen). Electroporation of the PCR products into these cells was performed; cells were incubated at 22°C for 16 h in SOC, and plated on media containing 30 µg ml−1 chloramphenicol overnight at 42°C. Resulting colonies were patched for chloramphenicol resistance and ampicillin sensitivity, and PCR-verified for the absence of the gene. The chloramphenicol cassette was then resolved from the mutants in order to create non-polar, isogenic *kdpE* and *qseB* mutants. Plasmid pCP20, encoding a resolvase, was electroporated into the mutant strains, and resulting colonies were patched for chloramphenicol sensitivity. Construction of *qseC* and *qseF* mutants has been previously published [7],[35].

### Site-directed mutagenesis

Site-directed mutagenesis was carried out using the Quick Change II site-directed mutagenesis kit (Stratagene). Mutagenesis PCR primers were constructed using the Primer X software (http://www.bioinformatics.org/primerx/) and are listed in Table 1 (qseBD51AF and qseBD51AR). The plasmid pVS154 was PCR amplified with the mutagenesis primers according to Stratagene's PCR protocol, generating the plasmid pDH12 (86-24 *qseB* D51A in pBADMycHis). The PCR product was digested with DpnI for 3 h at 37°C in order to remove the template plasmid. After digestion, the PCR product was transformed into XL-1 Blue supercompetent cells (Stratagene) and plated on selective media. The next day, plasmid DNA was isolated and sequenced to determine if the mutation was present.

### RNA extraction and real-time RT-PCR studies

Cultures were grown aerobically in LB medium at 37°C overnight, diluted 1∶100 in LB or DMEM (in the presence of self produced AI-3 and in the absence or presence of 10 µM epinephrine) and grown aerobically at 37°C. 0.2% arabinose was added to the media when induction was required. RNA from three biological replicate cultures of each strain was extracted at the late exponential growth phase (OD_600_ of 1.0) using the RiboPure Bacteria RNA isolation kit (Ambion) according to the manufacturer's guidelines. The primers used in the real-time assays were designed using Primer Express v1.5 (Applied Biosystems) (Table S2). Real-time reverse transcription-PCR (RT-PCR) was performed in a one-step reaction using an ABI 7500 sequence detection system (Applied Biosystems). For each 20-µl reaction mixture, 10 µl 2× SYBR master mix, 0.1 µl Multi-Scribe reverse transcriptase (Applied Biosystems), and 0.1 µl RNase inhibitor (Applied Biosystems) were added. Amplification efficiency of each of the primer pairs was verified using standard curves of known RNA concentrations. Melting-curve analysis was used to ensure template specificity by heating products to 95°C for 15 s, followed by cooling to 60°C and heating to 95°C while monitoring fluorescence. Once the amplification efficiency and template specificity were determined for each primer pair, relative quantification analysis was used to analyze the unknown samples using the following conditions for cDNA generation and amplification: 1 cycle at 48°C for 30 min, 1 cycle at 95°C for 10 min, and 40 cycles at 95°C for 15 s and 60°C for 1 min. The *rpoA* (RNA polymerase subunit A) gene was used as the endogenous control. Real-time RT-PCR primers for the LEE genes and *rpoA* have been previously described [33].

### Electrophoretic mobility shift assay (EMSA)

In order to study the binding of QseB to the *flhDC* promoter EMSAs were performed using the purified QseB protein and the *flhDC* promoter. DNA probes were then end-labeled with [γ-32P]-ATP (NEB) using T4 polynucleotide kinase using standard procedures [48]. End-labeled fragments were run on a 5% polyacrylamide gel, excised and purified using the Qiagen PCR purification kit. Electrophoretic mobility shift assays were performed by adding increasing amounts of purified QseB or QseBD51A protein (0–20 µM) to end-labeled probe (10 ng) in binding buffer [500 µg ml^−1^ BSA (NEB), 50 ng µl^−1^ poly-dIdC, 60 mM HEPES pH 7.5, 5 mM EDTA, 3 mM DTT, 300 mM KCl, 25 mM MgCl2] with or without 0.1 M acetyl phosphate for 20 min at 4°C. Immediately before loading, a 5% ficol solution was added to the mixtures. The reactions were electrophoresed for approximately 14 h at 65 V on a 5% polyacrylamide gel, dried and exposed to KODAK X-OMAT film.

#### Microarrays

Microarrays and analysis were performed as previously described [50]. The GeneChip *E. coli* Genome 2.0 array system of the Affymetrix system was used to compare the gene expression in strain 86-24 to that in strains VS138, MC474, and DH11. The GeneChip *E. coli* Genome 2.0 array includes approximately 10,208 probe sets for all 20,366 genes present in the following four strains of E. coli: K-12 lab strain MG1655, uropathogenic strain CFT073, O157:H7 enterohemorrhagic strain EDL933, and O157:H7 enterohemorrhagic strain Sakai (http://www.affymetrix.com/products/arrays/specific/ecoli2.affx). The RNA-processing, labeling, hybridization, and slide-scanning procedures were preformed as described in the Affymetrix Gene Expression Technical Manual (http://www.affymetrix.com/support/technical/manual/expression_manual.affx).

The output from scanning a single replicate of the Affymetrix GeneChip *E. coli* Genome 2.0 array for each of the biological conditions was obtained using GCOS v 1.4 according to the manufacturer's instructions. Data were normalized using Robust Multiarray analysis at the RMAExpress website (http://rmaexpress.bmbolstad.com/). The resulting data were compared to determine features whose expression was increased or decreased in response to inactivation of the *qseC*, *qseB*, *qseF* and *kdpE* genes. Custom analysis scripts were written in Perl to complete multiple array analyses. The results of the array analyses were further confirmed using real-time RT-PCR as described. We note that the isolate used in these studies has not been sequenced and thus is not fully contained on the array and that differences in genome content are evident. Expression data can be accessed using accession number (GSE15050) at the NCBI GEO database.

### Motility assays

Assays were performed as previously described [31]. Briefly, motility assays were performed at 37°C on 0.3% agar plates containing Tryptone media (1% tryptone and 0.25% NaCl). The motility halos were measured at 4 h and 8 h.

### Protein purification

One liter of LB media was inoculated at 1∶100 and grown to O.D. 0.6 at 30°C. The culture temperatures were reduced to 25°C, induced with 400 µM IPTG (Sigma) or 0.2% arabinose, and grown for either 3 h or 18 h. Cells were harvested, suspended in lysis buffer (50 mM phosphate buffer pH 8, 300 mM NaCl, and 20 mM imidazole) and lysed by homogenization. The lysed cells were centrifuged and the lysates were loaded onto to a Ni^2+^- NTA-agarose gravity column (Qiagen). The column was washed with lysis buffer and protein was eluted with elution buffer (50 mM phosphate buffer pH 8, 300 mM NaCl, 250 mM imidazole). Fractions containing purified protein were confirmed by SDS-PAGE and concentrated for further use.

### Reconstitution of QseC-His into liposomes

Liposomes were reconstituted as described previously [4],[51]. Briefly, 50 mg of *E. coli* phospholipids (20 mg/ml in chloroform; Avanti Polar Lipids) were evaporated and then dissolved into 5 ml of potassium phosphate buffer containing 80 mg of N-octyl-β-d-glucopyranoside. The solution was dialyzed overnight against potassium phosphate buffer. The resulting liposome suspension was subjected to freeze–thaw in liquid N2. Liposomes were then destabilized by the addition of 26.1 mg of dodecylmaltoside, and 0.625 mg of QseC-MycHis was added, followed by stirring at room temperature for 10 min. Two hundred-sixty milligrams of Biobeads (Biorad) were then added to remove the detergent, and the resulting solution was allowed to incubate at 4°C for 16 h. The supernatant was then incubated with fresh Biobeads for 1 h at 22°C the next day. The resulting liposomes containing reconstituted QseC-MycHis were frozen in liquid N2 and stored at −80°C until used.

### Autophosphorylation and phosphotransfer assays

Assays were performed as previously described [4]. Briefly, twenty microliters of the liposomes containing QseC-MycHis were adjusted to 10 mM MgCl2 and 1 mM DTT, and 10 µM epinephrine, frozen and thawed rapidly in liquid N2, and kept at room temperature for 1 h (this allows for the signals to be loaded within the liposomes). [γ32P]dATP (0.625 µl) (110 TBq/mmol) was added to each reaction. To some reactions, 12.5 µg of response regulator was added. At each time point (0, 10, 30 min), 10 µl of SDS loading buffer (with 20% SDS, to completely denature the liposome) was added. For all experiments involving QseC alone, a time point of 10 min was used. The samples were run on SDS/PAGE without boiling and visualized via PhosphorImager. The bands were quantitated by using imagequant version 5.0 software (Amersham Pharmacia).

### β-galactosidase assays

Assays were performed as previously described [31]. Briefly, bacteria containing *lacZ* fusions were grown overnight at 37°C in LB containing the appropriate selective antibiotic. Cultures were diluted 1∶100 and grown in LB, and when necessary supplemented with 0.2% arabinose, to an OD_600_ of 1.0 at 37°C. These cultures were then assayed for β-galactosidase activity using o-nitrophenyl-beta-d-galactopyranoside (ONPG) as a substrate as described previously [52].

## Supporting Information

Table S1 Strains and Plasmids.
(0.71 MB DOC)

Table S2 Oligonucleotide Primers.
(0.36 MB DOC)
