# Supplementary material for: The QseC Adrenergic Signaling Cascade in Enterohemorrhagic E. coli (EHEC)
Source: PLoS Pathog. 2009 Aug 21;5(8):e1000553. doi: 10.1371/journal.ppat.1000553 (PMC2726761; doi:10.1371/journal.ppat.1000553)
Supplement: Table S1 — Strains and Plasmids. (0.71 MB DOC) [file ppat.1000553.s001.doc]

**Table S1.** Strains and Plasmids

| Strains |  |  |
| --- | --- | --- |
| 86-24 | Wild-type EHEC strain (serotype O157:H7) | [1] |
| DH5α | *supE44 lacU169 (80 lacZ M15) hsdR17 recA1 endA1 gyrA96 thi-1 relA1* | Stratagene |
| VS138 | *qseC* mutant in 86-24 | [2] |
| VS179 | VS138 complemented with pVS178 | [2] |
| DH11 | *kdpE* mutant in 86-24 | this study |
| NR01 | *qseF* mutant in 86-24 | [3] |
| MC474 | *qseB* mutant in 86-24 | this study |
| MC484 | MC747 complemented with pVS154 | this study |
| MC550 | VS138 with pVS154 | this study |
| MC471 | Single-copy flhDC::lacZ (+50 bp to −900 bp) in MC1000 | [4] |
| DH13 | MC471 with pVS154 | this study |
|  |  |  |
| Plasmids |  |  |
| pBADMycHis | C-terminal Myc-His-Tag vector | Invitrogen |
| pBAD33 | Cloning vector | [5] |
| pVS178 | 86-24 *qseBC* in pBAD33 | [2] |
| pVS154 | 86-24 *qseB* in pBADMycHis | [6] |
| pVS155 | MC1000 *qseC* in pBADMycHis | [2] |
| pKD46 | λRed helper plasmid | [7] |
| pKD3 | λRed template plasmid | [7] |
| pCP20 | λRed resolvase plasmid | [7] |
| pDH12 | 86-24 *qseB* D51A in pBADMycHis | this study |

**References**

1. Griffin PM, Ostroff SM, Tauxe RV, Greene KD, Wells JG, et al. (1988) Illnesses associated with Escherichia coli O157:H7 infections. A broad clinical spectrum. Ann Intern Med 109: 705-712.

2. Sperandio V, Torres AG, Kaper JB (2002) Quorum sensing Escherichia coli regulators B and C (QseBC): a novel two-component regulatory system involved in the regulation of flagella and motility by quorum sensing in E. coli. Mol Microbiol 43: 809-821.

3. Reading NC, Torres AG, Kendall MM, Hughes DT, Yamamoto K, et al. (2007) A novel two-component signaling system that activates transcription of an enterohemorrhagic Escherichia coli effector involved in remodeling of host actin. J Bacteriol 189: 2468-2476.

4. Clarke MB, Sperandio V (2005) Transcriptional regulation of flhDC by QseBC and sigma (FliA) in enterohaemorrhagic Escherichia coli. Mol Microbiol 57: 1734-1749.

5. Guzman LM, Belin D, Carson MJ, Beckwith J (1995) Tight regulation, modulation, and high-level expression by vectors containing the arabinose PBAD promoter. J Bacteriol 177: 4121-4130.

6. Clarke MB, Sperandio V (2005) Transcriptional autoregulation by quorum sensing Escherichia coli regulators B and C (QseBC) in enterohaemorrhagic E. coli (EHEC). Mol Microbiol 58: 441-455.

7. Datsenko KA, Wanner BL (2000) One-step inactivation of chromosomal genes in Escherichia coli K-12 using PCR products. Proc Natl Acad Sci U S A 97: 6640-6645.
