# Supplementary material for: The QseC Adrenergic Signaling Cascade in Enterohemorrhagic E. coli (EHEC)
Source: PLoS Pathog. 2009 Aug 21;5(8):e1000553. doi: 10.1371/journal.ppat.1000553 (PMC2726761; doi:10.1371/journal.ppat.1000553)
Supplement: Table S2 — Oligonucleotide Primers. (0.36 MB DOC) [file ppat.1000553.s002.doc]

**Table S2.** oligonucleotide primers

| Name | Oligo |
| --- | --- |
| kdpEλRed-F | 5'-TTTTCGTGTTACACTTCCCCAGCAAACTGCCCCTGAACTT  GAAGAATTTCATGAGGATATGTGTAGGCTGGAGCTGCTTC-3' |
| kdpEλRed-R | 5'-ATTTGGCGCAGGTTTAATAATAAATTAATCACTATTTAG  GCGAATTTATTGAATAAAAATCATATGAATATCCTCCTTAG-3' |
| flhDrtF | 5'-TTTCGTCTCGGCATAAATGAAG-3' |
| flhDrtR | 5'-TCATTCAGCAAGCGTGTTGAG-3' |
| nleArt549F | 5'-AGCCACTACTTCGACGGTAACC-3' |
| nleArt624R | 5'-ACGAACCACTTGAGCTGTTAATCC-3' |
| stx2ArtF | 5'-ACCCCACCGGGCAGTT-3' |
| stx2ArtR | 5'-GGTCAAAACGCGCCTGATA-3' |
| recArtF | 5'-CAGGCGCGTGGTACAGCTA-3' |
| recArtR | 5'-CAGCCAGGCAGTTGCATTC-3' |
| kdpArtF | 5'-GCAGCATCAATATGGAAGGTAAAGA-3' |
| kdpArtR | 5'-ACGACCGCAAACAGGTACTGA-3' |
| qseBD51AF | 5’-GATGCGGTGATCCTGGCTTTAACCTTACCAGG-3’ |
| qseBD51AR | 5’-CCTGGTAAGGTTAAAGCCAGGATCACCGCATC-3’ |
| qseBλRed-F | 5’ -GTCCTTAACAACTTCTTAAGGGAAAAAAATAAAATT  TAGTGCTGTACAGAGCGCGTTACAACACGGTTTACTG  GCAGCGTGTAGGCTGGAGCTGCTTCG - 3’ |
| qseBλRed-R | 5’ -AAAAGATTAGCGTCAGCCTGACGCGCAGACTAAGAC  GTTGGGTAAATTTCATTTCTCACCTAATGTGTAACCAATA  CCATGCACCATATGAATATCCTCCTTA - 3’ |
